# Quinone and Hydroquinone Metabolites from the Ascidians of the Genus *Aplidium*

**DOI:** 10.3390/md12063608

**Published:** 2014-06-12

**Authors:** Camila Spereta Bertanha, Ana Helena Januário, Tavane Aparecida Alvarenga, Letícia Pereira Pimenta, Márcio Luis Andrade e Silva, Wilson Roberto Cunha, Patrícia Mendonça Pauletti

**Affiliations:** Center for Research in Exact and Technological Sciences, University of Franca, Avenue Doutor Armando Salles Oliveira, 201, Franca, São Paulo 14404-600, Brazil; E-Mails: camila_spereta@hotmail.com (C.S.B.); ana.januario@unifran.edu.br (A.H.J.); farmaciatata@hotmail.com (T.A.A.); leticia_pimenta94@hotmail.com (L.P.P.); marcio.silva@unifran.edu.br (M.L.A.S.); wilson.cunha@unifran.edu.br (W.R.C.)

**Keywords:** ascidian secondary metabolites, structure elucidation, bioactivity, ^13^C-NMR spectral data, quinones, hydroquinones

## Abstract

Ascidians of the genus *Aplidium* are recognized as an important source of chemical diversity and bioactive natural products. Among the compounds produced by this genus are non-nitrogenous metabolites, mainly prenylated quinones and hydroquinones. This review discusses the isolation, structural elucidation, and biological activities of quinones, hydroquinones, rossinones, longithorones, longithorols, floresolides, scabellones, conicaquinones, aplidinones, thiaplidiaquinones, and conithiaquinones. A compilation of the ^13^C-NMR spectral data of these compounds is also presented.

## 1. Introduction

Ascidians or tunicates, are the source of didemnin B, aplidine, and ecteinascidin 743, which have reached clinical trials as antitumor agents. Moreover, aplidine was isolated from *Aplidium albicans*. Thus, there is a high potential for these organisms to be a novel source of antitumor compounds [1,2]. In addition, the compound trabectedin, or ecteinascidin 743 (ET-743, Yondelis^®^, Toronto, Canada), an alkaloid isolated from the ascidian *Ecteinascidia turbinate*, was the first marine-derived drug to be prescribed against soft tissue carcinoma [3].

The name “tunicate” (sub-phylum Tunicata, Phylum Chordata) was first coined by Lamarck for ascidians, pyrosomes, and salps [4,5,6]. The name originates from the polysaccharide-containing tunic that envelops the animal and forms a somewhat flexible skeleton [7]. Ascidians (Subphylum Tunicata, Class Ascidiacea), or sea squirts, are the largest and most diverse class of the sub-phylum Tunicata (also known as Urochordata). They comprise approximately 3000 described species, found in all marine habitats, from shallow water to the deep sea [7,8,9]. Polyclinidae is one of the most diverse families in the class Ascidiacea, within which *Aplidium* is the most species-rich genus, with 320 known species, based on the species registered in the online World Register of Marine Species [10]. Ascidians, which belong to the family Polyclinidae, genus *Aplidium*, have been the subject of extensive chemical and biological investigations [11].

The natural products isolated from *Aplidium* species can be classified into two groups: nitrogenous and non-nitrogenous compounds. Numerous non-nitrogen containing metabolites have been isolated, mainly linear or cyclic prenyl hydroquinones and quinones, which are known as meroterpenes and present cytotoxic activities [3,11,12]. Although, there are examples of metabolites that contains nitrogen in the prenylated quinone moiety, such as conicaquinone A, which has an unusual 1,1-dioxo-1,4-thiazine ring added to the quinone moiety. These meroterpenes are the focus of the current review, which describes the structures, biological activities and ^13^C-NMR data of 53 prenylated hydroquinones and quinones isolated from *Aplidium*, thus, highlighting the structural diversity generated in this class of natural products and their potential in drug discovery. A large number of compounds (48) have been examined by ^13^C-NMR spectroscopy so considerable ^13^C chemical shift data have accumulated; however, a compilation of ^13^C data for meroterpenes from *Aplidium* has not been available to date. The assignment of carbon from known compounds is simple and straightforward, providing ^13^C data of appropriate model compounds are available. Thus, we provide a tabulation of ^13^C-NMR data to provide easy access to previously published data on meroterpenes. This paper reports also a brief description of the most characteristics ^1^H-NMR chemical shift data.

Prenyl quinones and hydroquinones are metabolites of mixed biogenesis that originate from intra- and inter-molecular cyclizations and/or rearrangements, thus, producing macrocyclic or polycyclic skeletons that are often linked to amino acids or taurine residues [11]. In this report, we have focused on quinones and hydroquinones from Ascidians of the genus *Aplidium* and their related compounds: rossinones, longithorones, longithorols, floresolides, scabellones, conicaquinones, aplidinones, thiaplidiaquinones, and conithiaquinones. The described compounds were isolated from organisms belonging to 10 identified and three unidentified species of *Aplidium*. The determination of their structures and biological activities are also summarized, and a compilation of ^13^C-NMR spectral data is provided. The intent of the present review is to provide the researchers who have isolated a prenyl quinones and hydroquinones from *Aplidium* with a quick means of deciding whether the compound is known or new, and to allow them to establish a structure by comparison of ^13^C-NMR data. Additionally, ^1^H-NMR spectroscopic studies are often sufficient to establish structures unequivocally; although, there are isomeric molecules that ^13^C-NMR data were important to resolve structure problems. *Aplidium* are clearly prolific producers of bioactive prenylated quinones and hydroquinones in the marine environment, and many other *Aplidium* species need to be investigated.

### 1.1. Quinones

**Figure 1:**
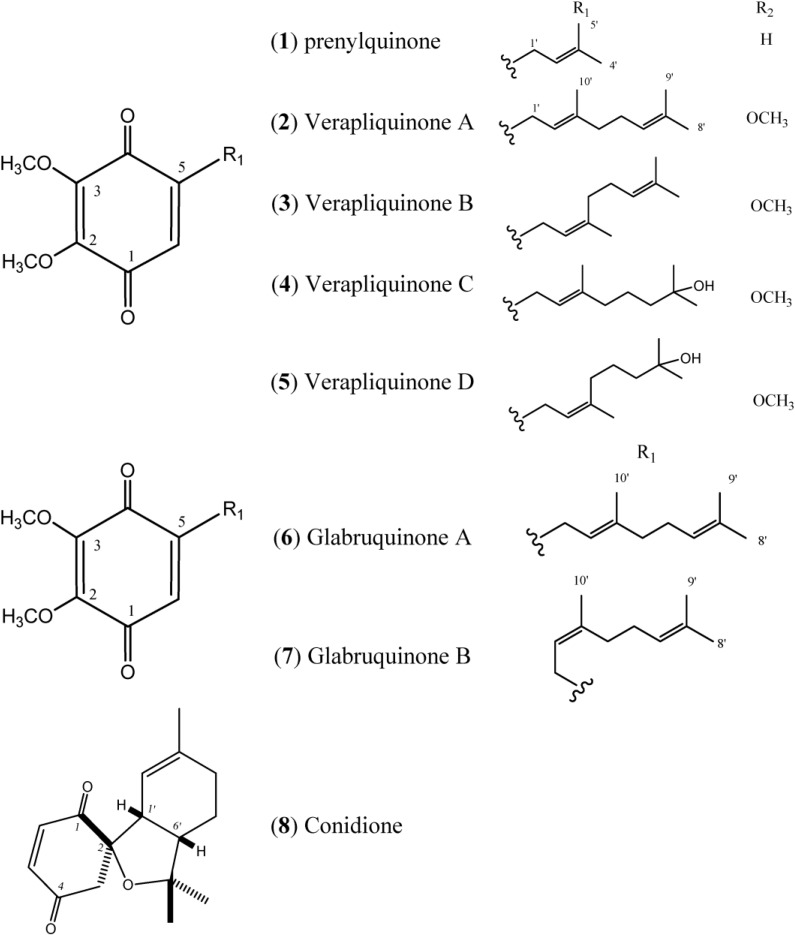
Structures of Quinones (**1**–**8**).

Quinones can be derived by the oxidation of appropriate phenolic compounds, with 1,2-dihydroxybenzenes and 1,4-dihydroxybenzenes, yielding *ortho*-quinones and *para*-quinones respectively. Therefore, quinones can be formed from phenolics compounds by either the acetate or shikimate pathways, affording a catechol or quinol system. A range of quinone derivatives and related structures that contain a terpenoid fragment or shikimate-derived portion are also wide spread. For example, ubiquinones (coenzyme Q) have important biochemical functions in electron transport systems for respiration [13]. Figure 1 describes quinones that occur in *Aplidium*.

The simple linear Prenylquinone (**1**) was isolated from *Aplidium californicum* [14]. The verapliquinones A–D (**2**–**5**) were isolated from an unidentified *Aplidium* sp. (Ascidiacea) collected off the Breton coast. Verapliquinones A/B and C/D were characterized in a mixture of *E*,*Z*-isomers by NMR spectroscopy. The NMR data revealed that Verapliquinone B and D had a neryl group at C-2 instead of geranyl [15]. Davis and co-workers [16] reported a simple and versatile route to 1,4-benzoquinones based on the Claisen rearrangement, and applied to the synthesis of verapliquinones A and B, which had not previously been synthesized [16]. Chan and co-workers (2011) also reported verapliquinone A from *A. scabellum* [17].

Glabruquinone A (**6**) (or desmethylubiquinone Q_2_) and Glabruquinone B (**7**) were isolated from *Aplidium glabrum* and synthesized. The main difference between **6** and **7** is that compound **6** contains a geranyl side chain instead of neryl as in **7** [18]. Glabruquinone A (**6**) displayed cancer preventive activity in the anchorage-independent transformation assay against mouse JB6 P^+^ Cl 41 cells transformed with an epidermal growth factor, with an inhibition of the number of colonies C_50_ (INCC_50_) value of 7.3 µM. The INCC_50_ values for **6** were 12.7, 17.5, and 50.5 µM against HCT-116, MEL-28, and HT-460 human tumor cells, respectively. Compound **6**, at 10 µM, increased the UVB-induced p53 transcriptional activity of JB6 P^+^ Cl 41 cells [18,19]. Glabruquinone A was also evaluated *in vivo* on mice inoculated with Ehrlich carcinoma tumors and found to inhibit tumorgrowth. Compound **6** inhibited the phenotype expression of HT-460, HCT-116, and SK-MEL-28 human tumor cells and induced apoptosis of these cell lines, as well as that of HL-60 and THP-1 tumor cells [20].

Conidione (**8**), a cyclic diprenyl quinone, was isolated from *A. conicum* but was unstable, and this instability prevented bioassays from being performed [21].

### 1.2. Hydroquinones

**Figure 2:**
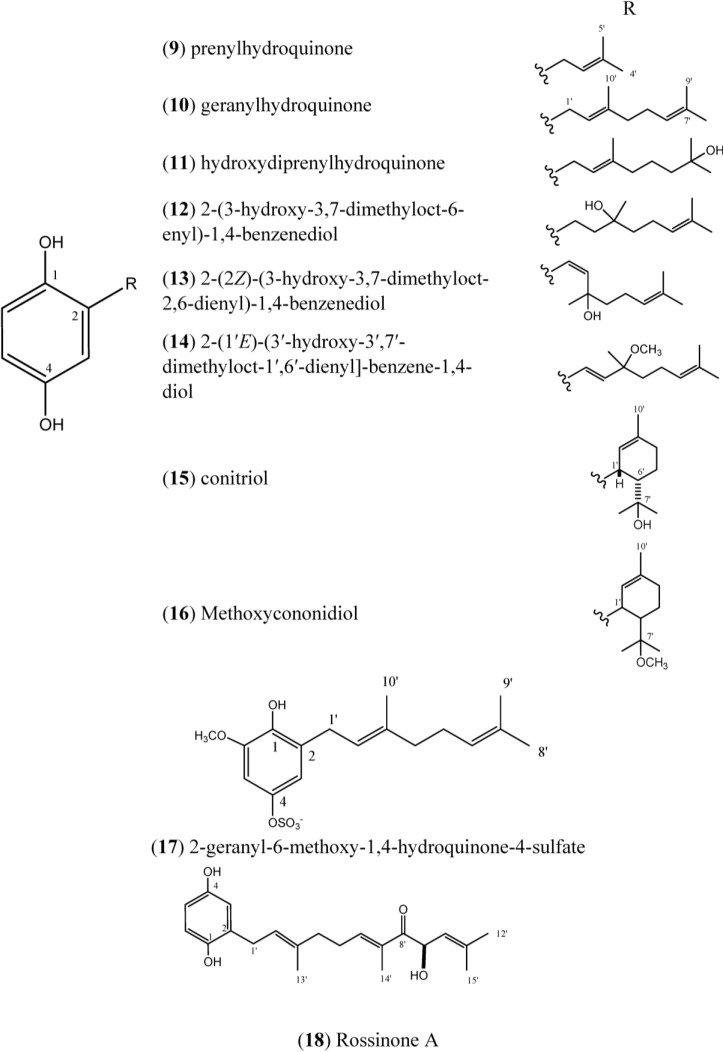
Structures of Hydroquinones (**9**–**18**).

Prenylhydroquinone (**9**), Figure 2, isolated from *A. californicum*, exhibited activity *in vivo* against P388 lymphocytic leukemia. The potential cancer protective properties of prenylhydroquinone (**9**) were also evaluated by employing a modified-Ames assay for mutagenicity against *Salmonella typhimurium*; when prenylhydroquinone was added to experiments, the mutagenic effects of the carcinogens were drastically reduced [14].

Compound **9** was able to form stable semiquinone radicals according to Cotelle and collaborators, and in presence of glutathione, **9** was involved in a redox cycle with the consumption of oxygen. This process triggered the formation of free radicals and decreased the glutathione content, which is considered to be one of the major defenses against oxidative damage. Even although not fully elucidated, the antitumor properties of **9** can be correlated with its redox properties and reactivity toward glutathione [22]. Prenylhydroquinone also inhibits superoxide anion production in rat alveolar macrophages and in the xanthine/xanthine oxidase system. The antioxidant activity of **9** may be attributed to a direct reaction of the superoxide anion rather than to an enzymatic inhibition or a membrane signal transfer [23].

Prenylhydroquinone (**9**) and geranylhydroquinone (**10**), isolated from *Aplidium* sp., have exibited antiproliferative activity (IC_50_: 41 and 9.5 μM, respectively) in a P388 murine leukemia cell-line. Geranylhydroquinone (**10**) at doses up to 30 μM was inactive against the solid tumor cell lines A375 (human melanoma), A549 (human breast), HepG2 (human hepatic), and HT-29 (human colon), as well as normal human liver cells (WRL-68) [24]. Both prenylhydroquinone (**9**) and geranylhydroquinone (**10**) exhibited anti-inflammatory activity in an *in vitro* anti-inflammatory assay with activated human peripheral blood neutrophils by inhibing superoxide production. Compound **10** was also tested in the DPPH radical scavenging assay, and was considered inactive [24].

Geranylhydroquinone (**10**) has been reported to occur in various species of *Aplidium*. It has been isolated from two unidentified *Aplidium* sp [25,26], *A. antillense* [27], *A. nordmani* [28], *A. savignyi* [29], and *A. coninum* [21]. Compound **10** has also been isolated from plants [30,31,32].

Geranylhydroquinone exhibited cytotoxicity against the leukemia cell lines of Rous sarcoma and mammary cincinoma *in vivo* [33]. Cytotoxic activity was also observed for **10** against P-388 leukemia (IC_50_ 0.034 µg/mL) and KB human epidermoid carcinoma cells (IC_50_ 4.3 µg/mL). Geranylhydroquinone (**10**) has also demonstrated antibacterial activity, with a minimum inhibitory concentration (MIC) of 64 µg/mL against *Staphylococus aureus* and *S. faecalis*, and an MIC of 128 µg/mL against *Serratia marcescens*. The minimum bactericide concentrations (MBCs) were determined to be 2, 1, and ˃4 µg/mL, respectively [27]. Additionally, Compound **10** was more potent than two standard antioxidants in terms of its inhibitory effects on lipid peroxide formation in rat liver microsomes and on soy-bean 15-lipoxygenase [34].

Geranylhydroquinone (**10**) and hydroxydiprenylhydroquinone (**11**) displayed significant cytotoxicity against four tumor cell lines, in particular, against P-388 mouse lymphoma suspension culture (IC_50_ 0.81 and 4.5 µM, respectively) indicating that the hydroxylation of the prenyl chain in **11** may result in a marginal decrease in its cytotoxicity [26].

The compound 2-(3ʹ-hydroxy-3ʹ,7ʹ-dimethyloct-6ʹ-enyl)-1,4-benzenediol (**12**) was isolated from de *A. conicum* and *A. savignyi* [14,22]. The compound 2-(2ʹ*Z*)-(3ʹ-hydroxy-3ʹ,7ʹ-dimethyloct-2ʹ,6ʹ-dienyl)-1,4-benzenediol (**13**) was isolated from *A. savignyi* [29].

The compounds 2-(1ʹ*E*)-(3ʹ-hydroxy-3ʹ,7ʹ-dimethyloct-1ʹ,6ʹ-dienyl]-benzene-1,4-diol (**14**) and conitriol (**15**) were isolated from *A. conicum* but exhibited chemical instability. This instability prevented pharmacological assays from being conducted [21].

Methoxyconidiol (**16**) was isolated from *A. aff. densum* [35] and its effects were tested on sea urchin embryos during cell division; compound **16** disturbed the mitotic spindle assembly leading to a cell cycle arrest during the metaphase/anaphase transition [36]. The antibacterial activity of **16** on *Escherichia coli* and *Micrococcus luteus* was also evaluated by microtiter broth dilution method; it had no antibacterial effect [37]. Methoxyconidiol inhibited egg division, with IC_50_ values of 0.80 μM for *Paracentrotus lividus* eggs and 4.30 μM for *Sphaerechinus granularis* eggs. When tested against the human carcinoma cell lines: MCF7 (breast), PA1 (ovary), PC3 (prostate), CEM-WT (acute lymphoblastic leukemia), and L-929 murine immortalized cells, as well as normal human fibroblasts, **16** was non-toxic to all these cell lines with an IC_50_ > 100 µM [35,37]. Methoxyconidiol caused antimitotic action during the first division of sea urchin embryos, and the mechanism of action of methoxyconidiol may be mediated by the disruption of microtubule dynamics [29]. Hence, Simon-Levert *et al.* 2010 [37] concluded that methoxyconidiol was ineffective against human cancer cells but effective in sea urchin cells. This finding could be explained by a difference between these two type of cells in either membrane permeability and/or intracellular transport of **16**.

The compound 2-geranyl-6-methoxy-1,4-hydroquinone-4-sulfate (**17**) was isolated from *A. scabellum* and inhibited superoxide production by PMA-stimulated human neutrophils *in vitro*, with an IC_50_ of 21 μM. To determine the effect of different treatments on cell survival, drug-treated neutrophils were stained with the fluorescent markers for necrosis (propidium iodide) and apoptosis (Annexin V-FITC), and analyzed by flow cytometry. Treatment with **17** had no effect on neutrophil viability, but the results indicated that **17** did inhibit neutrophil superoxide production [17].

### 1.3. Rossinones

**Figure 3:**
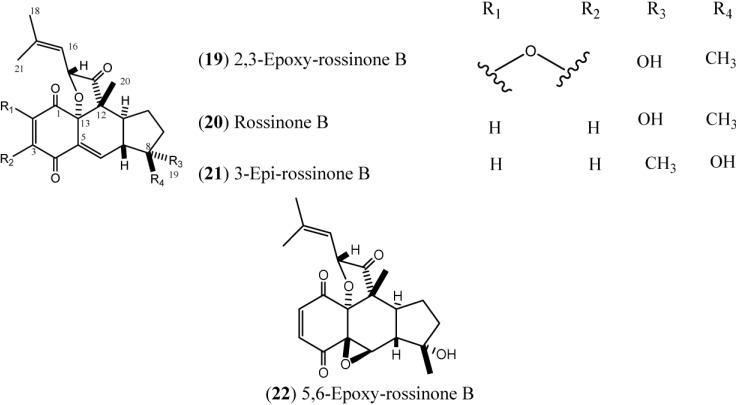
Structures of Rossinones (**19**–**22**).

Rossinones (Figure 3), particularly rossinone B (**20**) and its derivatives, are linearly fused in a 6,6,5-ring core of rossinone B, which is an extremely rare skeleton, known for only three plant-derived natural products. Rossinone B was isolated for the first time from an unidentified Antarctic species of *Aplidium* and then from *A. fuegiense* [24,38]. Therefore, the isolation of **20** extended the evolutionary range of the requisite biosynthetic terpene cyclase(s) from Plant kingdom to Animalia [24]. To date, five rossinones (**18**–**22**) have been discovered.

Rossinones A (**18**) and B (**20**) exhibited anti-inflammatory activity in an *in vitro* anti-inflammatory assay with activated human peripheral blood neutrophils by inhibiting superoxide production. However, in the DPPH radical scavenging assay, **18** and **20** were found to be inactive (at doses up to 30 μM), indicating that these rossinones are considerably less effective as superoxide scavengers than as suppressors of superoxide production by neutrophils. Rossinones A and B have exhibited selective antiviral activity against the DNA virus HSV-1, *versus* the RNA virus PV-1, with both compounds exhibiting antiviral activity at 2 μg/disk. Both compounds also exhibited antimicrobial activities against *Bacillus subtilis* and the fungus *Trichophyton mentagrophytes* [24].

Rossinone B (**20**) exhibited potent antiproliferative activity toward the P388 murine leukemia cell-line (IC_50_: 0.084 μM), while rossinone A (**18**) was less active (IC_50_: 0.39 μM). Rossinone A (**18**) was inactive against the solid tumor cell lines A375 (human melanoma), A549 (human breast), HepG2 (human hepatic), and HT-29 (human colon) at doses up to 30 μM, while rossinone B (**20**) presented good activity toward the SH-SY5Y neuroblastoma cell line (IC_50_: 1.6 μM) and modest activity against the A375, A549, and HT-29 cell lines with IC_50_ values of 11, 30, and 30 μM, respectively. Additionally, **18** and **20** exhibited antiproliferative activity against normal human liver cells (WRL-68) at concentrations up to 30 μM [24].

The rossinones 2,3-epoxy-rossinone B, rossinone B, 3-epi-rossinone B, and 5,6-epoxy-rossinone (**19**–**22**) isolated from *A. fuegiense*, mainly rossinone B was proven to be involved in the whole-colony chemical defense of *A. fuegiense*, repelling sea stars and amphipods [39].

### 1.4. Longithorones, Longithorols and Floresolides

**Figure 4:**
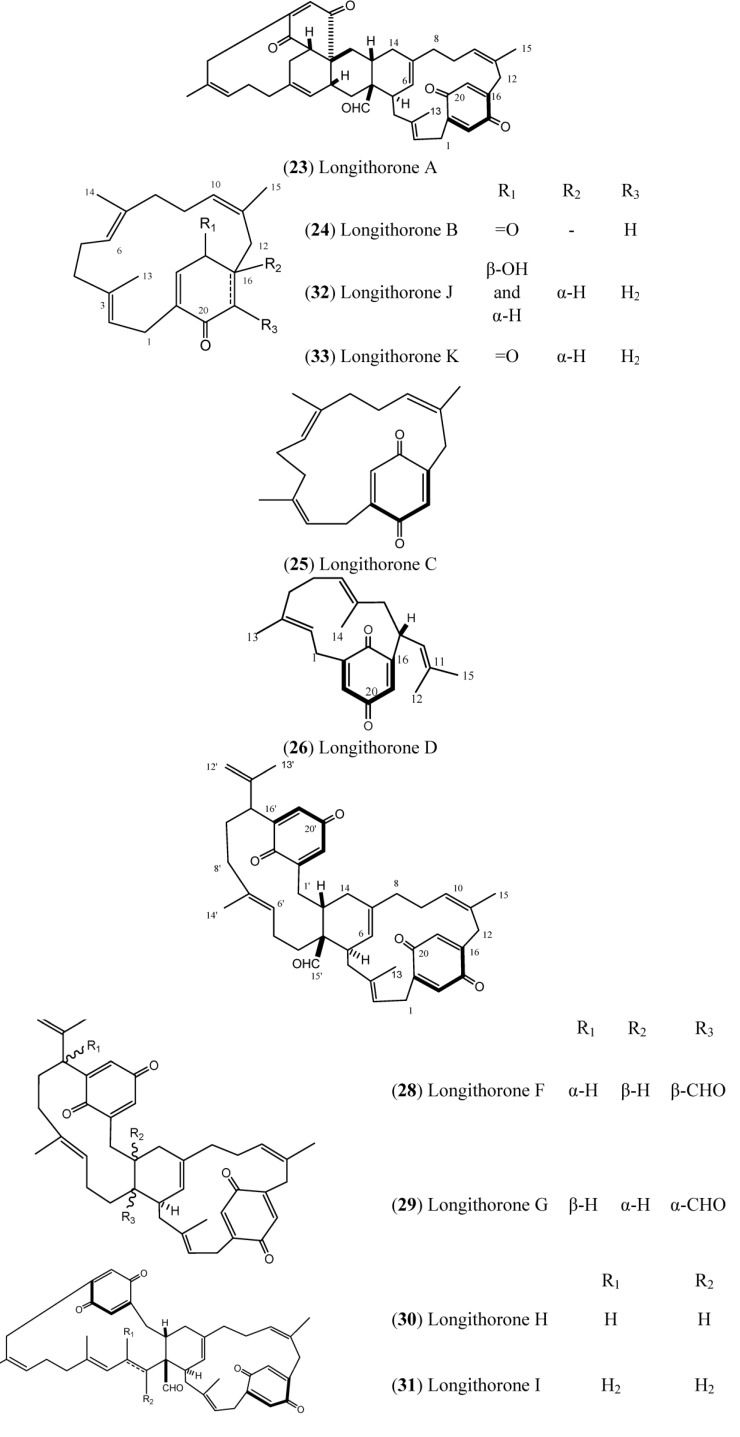
Structures of Longithorones (**23**–**33**).

**Figure 5:**
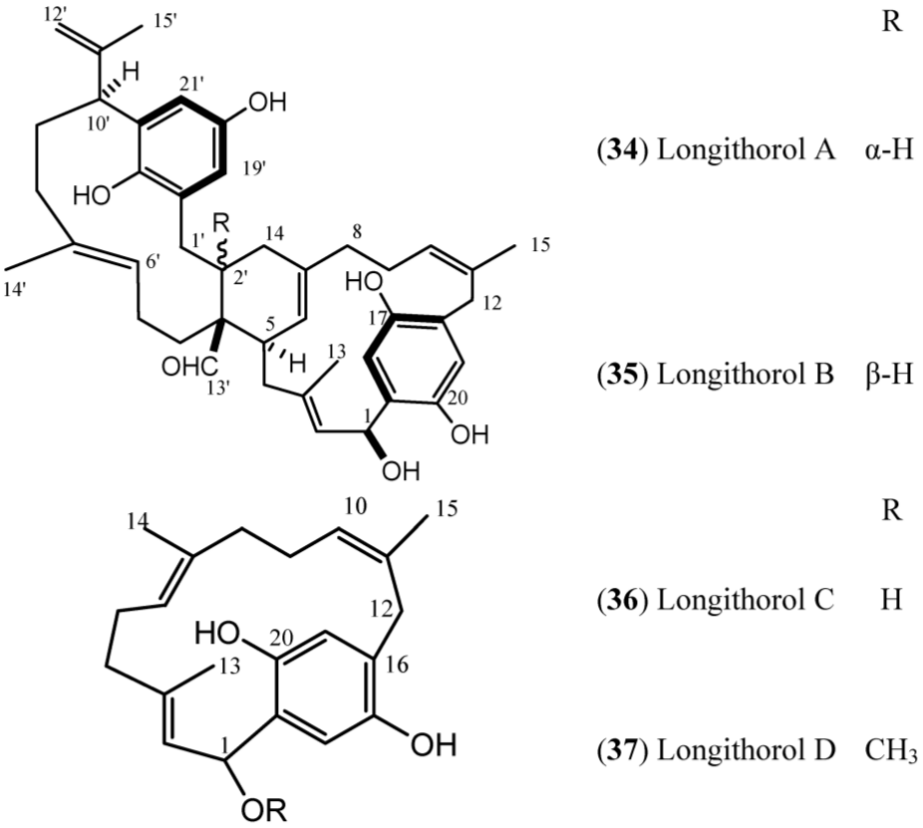
Structures of Longithorol (**34**–**37**).

Longithorones (Figure 4) and longithorols (Figure 5) are unique farnesylated quinones/hydroquinones isolated from *A. longithorax* (Monniot). Their complex structures are characterized by the presence of a metacycophane and/or paracyclophane system built in a farnesyl quinone or hydroquinone formally by the rarely encountered cyclization of farnesyl quinones/hydroquinones [12]. One special characteristic of those compounds is the atropisomerism that is caused by the restricted rotations of their macrocyclic rings. Eleven compounds have been isolated (longithorones A–K), including monomeric prenylated quinones (**24**–**26**) and dimeric prenylated quinones (**23** and **27**–**31**), and the cyclofarnesylated quinones (longithorones J and K; **32**–**33**) [40,41]. The biosynthesis of dimeric longithorones, which have been supposed to originate by both intra- and intermolecular Diels-Alder reactions, has been speculated about. Fusion of the two farnesyl-quinone units can be envisioned as arising via a Diels-Alder cycloaddition of suitably unsaturated precursors, whereas rings B and C could arise by a transannular Diels-Alder reaction. The co-isolation of the monomers provides some support for this proposal [12].

Longithorone A (**23**) displayed cytotoxicity against P388 murine leukemia cells with an IC_50_ of ~10 mg·mL^−1^ [42]. In addition, the longithorone J (**32**) was tested for cytotoxicity against the cell lines SHSY5Y (human neuroblastoma), HEK293T (SV40 T antigen transformed human embryonal kidney cells) and A549 (human non-small cell lung carcinoma). Compound **32** did not exhibit any cytotoxicity in the A549 cell assay when tested at 2 and 20 mg/mL. However, **32** displayed minimal activity at 20 mg/mL against the SHSY5Y and HEK293T cells, with cell deaths of 28% and 16%, respectively [43].

Zacarian and collaborators synthesized the highly rigid macrocyclic carbon skeleton of longithorone C (**25**) by exploiting quadrupolar interactions as synthetic strategy [44]. Despite the fact that cyclophanes have been extensively synthesized and evaluated because of their unique physical and chemical properties, examples of the isolation and total synthesis of cyclophane-containing natural products are rare and challenging. This has attracted the interest of synthetic chemists in search of new and efficient strategies for syntheses with a reduced number of steps [45,46].

Longithorols A (**34**) and B (**35**) are prenylated paracyclophane and metacyclophane hydroquinones, and longithorols C (**36**) and D (**37**) are para-substituted cyclofarnesylated hydroquinones. The hydroquinones (longithorols A–D, **34**–**37**) were also isolated from *A. longithorax*, moreover **34** and **35** were isolated as their pentaacetate forms because of their instability [47,48].

**Figure 6:**
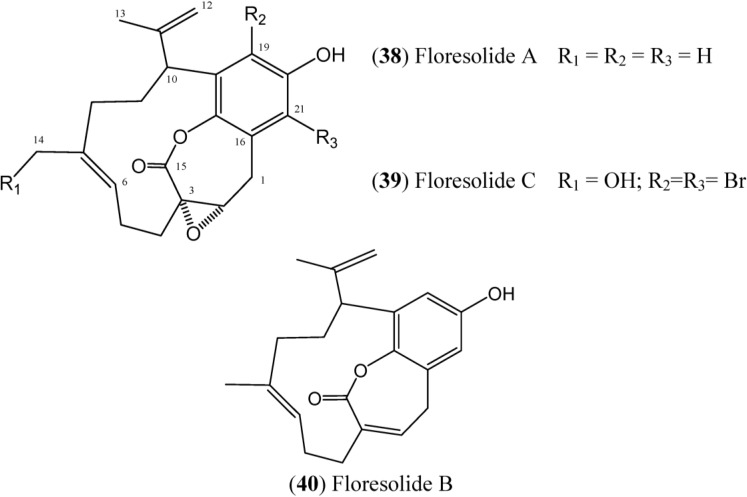
Structures of floresolides floresolides (**38**–**40**).

Floresolides are monomeric cyclofarnesylated hydroquinones with an endocyclic ε-lactone. They are members of longithorone/longithorol class of meroterpenes. Floresolides A–C (**38**–**40**) (Figure 6) have been isolated from *Aplidium* sp. collected in Indonesian. All of these floresolides exhibited moderate cytotoxicity against KB tumor cells [49]. The synthesis of floresolide B (**40**) hydroquinone lactone core was performed by employing ring-closing metathesis approach by Briggs and Dudley [50]. The total synthesis of racemic floresolide B was reported by Nicolaou and Xu, who used an olefin metathesis-based strategy for the formation of the macrocyclic lactone portion [51,52].

### 1.5. Scabellones

**Figure 7:**
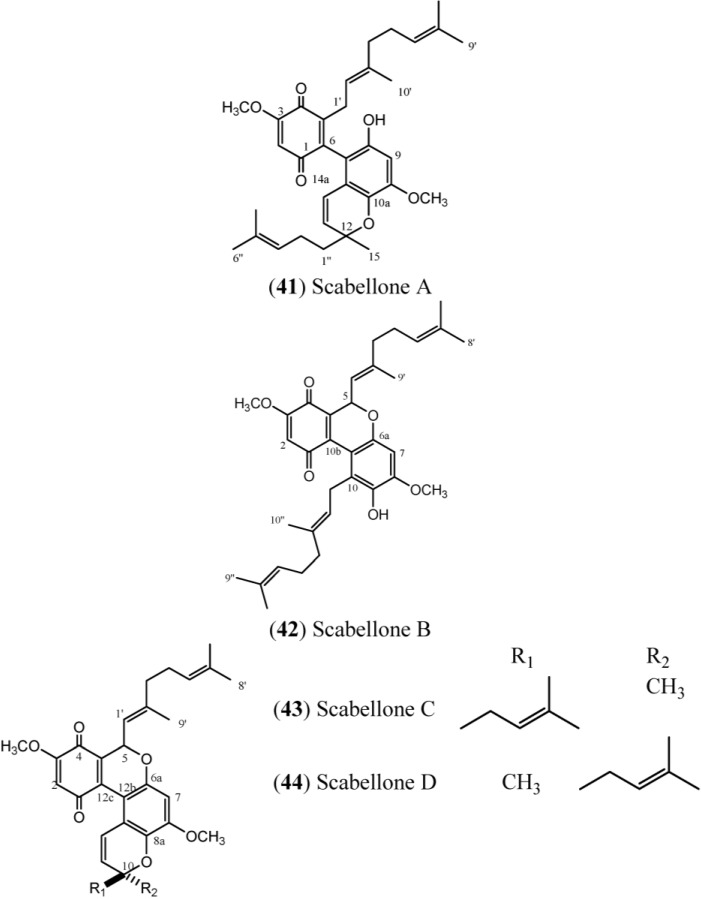
Structures of scabellones (**41**–**44**).

Chan *et al.*, 2011 [17], have described the isolation of the pseudodimeric meroterpenoid scabellones A–D (**41**–**44**) (Figure 7). Scabellone B (**42**) was able to inhibit the superoxide production by PMA-stimulated human neutrophils *in vitro*, with an IC_50_ of 125 μM. In contrast, **42** had no effect on neutrophil viability. Scabellone B (**42**) was also evaluated against the neglected disease parasite targets *Trypanosoma brucei rhodesiense, T. cruzi*, *Leishmania donovani*, and *Plasmodium falciparum*. This compound exhibited selectivity towards only *P. falciparum* (a K1 chloroquine-resistant strain), with an IC_50_ of 4.8 μM, and demonstrated poor cytotoxicity (in a L6 rat myoblast cell line, IC_50_: 65 μM). The core benzo[*c*] chromene-7,10-dione scaffold of scabellones A–D is rare among natural products and has previously been associated with antiproliferative or apoptosis-inducing biological properties.

### 1.6. Conicaquinones, Aplidinones, Thiaplidiaquinones and Conithiaquinones

**Figure 8:**
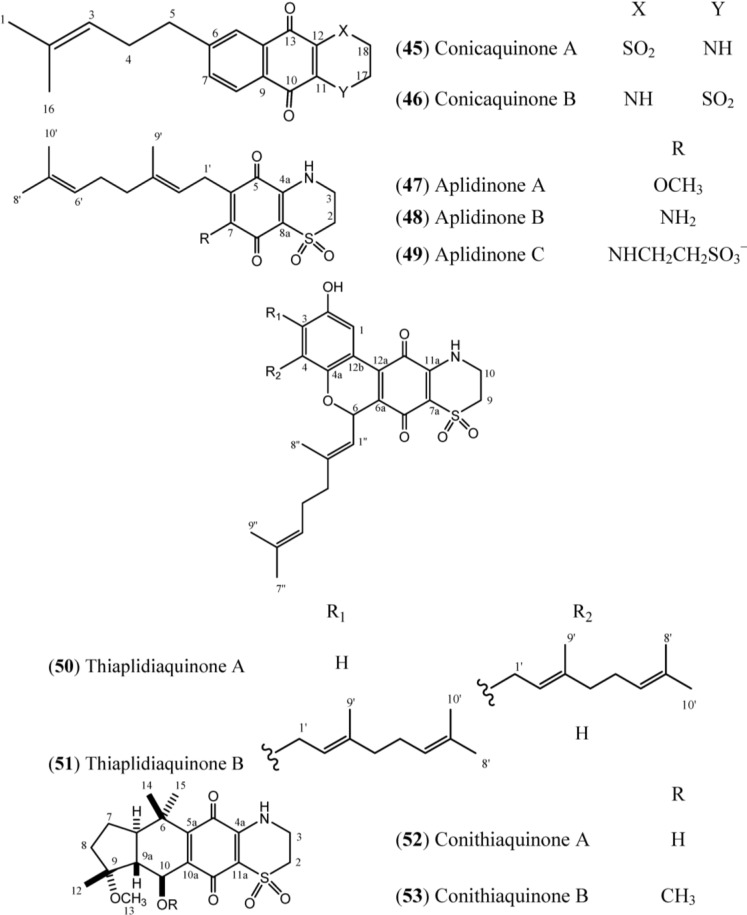
Structures of conicaquinones (**45**, **46**), aplidinones (**47**–**49**), thiaplidiaquinones (**50**, **51**), and conithiaquinones (**52**, **53**).

Two cytotoxic terpene quinones, the isomeric prenylated quinones conicaquinones A and B (**45**, **46**) (Figure 8), which have an unusual 1,1-dioxo-1,4-thiazine ring added to their quinone moiety, have been isolated from Mediterranean ascidian *A. conicum*. Both compounds were evaluated *in vitro* on rat glioma (C6) and rat basophilic leukemia (RBL-2H3) cell lines and demonstrated selectivity against rat glioma cells [53]. A follow-up study with this species, conducted by the same research group resulted in the isolation of three new geranylated quinones, aplidinones A–C (**47**–**49**) [54] and two new prenylated benzoquinones, designated as thiaplidiaquinones A and B (**50**, **51**), with an unprecedented tetracyclic skeleton in which a chromenol unit is attached to a *p*-benzoquinone ring condensed to a 1,4-thiazine-dioxide ring. Thiaplidiaquinones A and B were investigated for their antitumor activity. Both compounds were able to induce apoptosis in Jurkat cell lines that were derived from a human T lymphoma through the overproduction of reactive oxygen species (ROS), which mediated the collapse of the mitochondrial potential (ΔΨ_m_). The thiaplidiaquinones 50 and 51 exhibited cytotoxic activity against the human leukemia T cell line Jurkat cells, with an IC_50_ of approximately 3 μM [55]. The total synthesis of thiaplidiaquinone A (**50**) was described by Carbone [56], while the biomimetic synthesis of thiaplidiaquinone A and B was developed by Copp around the same time [57].

Two tetracyclic meroterpenes, conithiaquinones A and B (**52**, **53**), were isolated from *A. conicum* collected off the coast of Sardinia, Italy, and were assayed for their effect on the *in vitro* growth and viability of human skin keratinocyte (HaCaT line) and human breast adenocarcinoma (MCF-7 line) cells. Conithiaquinone A (**52**) demonstratedmoderate cytotoxicity against MCF-7 cells with an IC_50_ value of 44.5 μM [58].

### 1.7. Structural Elucidation

A compilation of the ^13^C chemical shifts of the quinones (**2**–**4** and **6**–**8**), hydroquinones (**9**–**12** and **14**–**17**), rossinones (**18**–**22**), longithorones (**23**–**29** and **32**–**33**), longithorols (**34**–**37**), floresolides (**38**–**40**), scabellones (**41**–**44**), conicaquinones (**45**–**46**), aplidinones (**47**–**49**), thiaplidiaquinones (**50**–**51**), and conithiaquinones (**52**–**53**), is given in Table 1, Table 2, Table 3, Table 4, Table 5, Table 6 and Table 7. The solvent (A = CDCl_3_, B = Me_2_CO-*d_6_*, C = C_6_D_6_, D = CD_3_OD, E = CD_2_Cl_2_ and F = DMSO-*d_6_*) and references are shown in the first line of the tables. Inspection of the ^13^C-NMR data of compounds **2**–**4** and **6**–**8** (prenylated quinones) compared with **9**–**12** and **14**–**17** (prenylated hydroquinones) (Table 1 and Table 2) reveals the introduction of hydroxyl groups in the C-1 and C-4 in **9**–**12** and **14**–**17** (instead of carbonyls group as in **2**–**4** and **6**–**8**) results in upfield signals at the α carbon. Thus, the quinone family skeleton can be recognized by the typical carbonyl signals.

**Table 1:** ^13^C chemical shifts (δ in ppm) of quinones derivatives. Ref. = References, n.d. = not detected.

Carbon	2	3	4	6	7	8
Solvent	A	A	A	A	A	A
Ref.	[15]	[15]	[15]	[18]	[18]	[21]
1	182.2	182.2	187.7	184.3	184.3	197.9
2	146.4	146.4	n.d.	144.9	144.9	86.9
3	132.8	132.8	132.8	145.1	145.1	50.4
4	187.7	187.7	187.7	184.5	184.5	196.6
5	107.1	107.1	107.1	146.9	146.9	141.1
6	158.9	158.9	158.9	130.4	130.4	140.7
1ʹ	27.1	26.9	27.2	27.1	27.2	41.8
2ʹ	117.7	118.4	117.9	117.7	117.7	116.2
3ʹ	140.0	140.0	139.9	140.1	140.1	139.0
4ʹ	39.6	31.8	39.9	39.7	32.0	29.2
5ʹ	26.4	26.3	22.5	26.5	26.5	21.9
6ʹ	123.8	123.8	43.4	123.9	123.9	46.0
7ʹ	131.8	131.8	70.9	131.9	131.9	84.7
8ʹ	25.6	25.6	29.3	25.7	25.7	24.2
9ʹ	17.7	16.1	29.3	17.7	17.7	23.7
10ʹ	16.1	23.5	16.0	16.2	22.7	29.0
OCH_3_	56.2	56.2	56.3	61.3	61.3	-
OCH_3_	-	-	-	61.2	61.2	-

The prenylated quinone family skeleton can also be recognized by the typical ^1^H-NMR signals; for example, in the case of compound **2**, the doublet of the triplet at δ_H_ 6.43 (H-3, *J* = 2.8 and 2.2 Hz) and a doublet at δ_H_ 5.86 (H-5, *J* = 2.8), and the presence of a methoxyl group at δ_H_ 3.80 s are typical of the pattern for a 2,6-substituted quinone nucleus [15]. On the other hand, the prenyl hydroquinone core (2-substituted hydroquinone) can be evinced by the typical ^1^H-NMR signals for three aromatic hydrogens corresponding to two with an *ortho* coupling at δ_H_ 6.62 (d, *J* = 8.5 Hz) and δ_H_ 6.52 (dd, *J* = 8.4 and 2.85 Hz) and one with a *meta* coupling at δ_H_ 6.55 (d, *J* = 2.5 Hz), as observed in **10** [29].

**Table 2:** ^13^C chemical shifts (δ in ppm) of hydroquinones derivatives.

Carbon	9	10	11	12	14a	15	16	17
Solvent	B	A	A	A	C	C	A	D
Ref.	[14]	[29]	[25]	[29]	[21]	[21]	[35]	[17]
1	148.3	148.6	147.8	147.9	147.3	148.6	149.4	141.2
2	129.3	147.7	138.2	123.8	135.9	130.8	130.5	129.2
3	116.9	116.6	121.8	116.6	113.2	118.7	118.8	114.1
4	150.8	148.2	149.7	148.9	150.3	148.8	148.4	146.4
5	113.5	114.3	116.5	113.8	115.2	114.2	114.3	103.6
6	116.1	116.2	116.4	116.9	116.8	118.4	118.2	148.6
1ʹ	28.9	30.0	29.0	41.4	125.2	34.3	34.4	29.1
2ʹ	123.6	120.8	113.6	24.5	124.5	125.4	125.6	123.9
3ʹ	132.1	128.1	128.1	73.8	77.6	133.7	133.8	136.7
4ʹ	17.5	40.0	42.9	41.7	40.6	31.5	31.8	41.0
5ʹ	-	26.7	22.4	22.9	23.0	20.7	20.6	27.9
6ʹ	-	123.6	39.7	124.0	n. d.	49.1	47.4	125.5
7ʹ	-	122.0	71.4	132.4	131.1	75.3	79.5	132.2
8ʹ	-	26.6	29.2	25.7	25.8	32.5	25.7	25.9
9ʹ	-	18.1	29.2	17.7	22.1	23.3	19.1	17.8
10ʹ	-	15.5	16.2	26.6	17.7	22.9	23.3	16.3
OCH_3_	-	-	-	-	49.9	-	49.1	56.6

**Table 3:** ^13^C chemical shifts (δ in ppm) of rossinones derivatives.

Carbon	18	Carbon	19	20	21	22
Solvent	A	Solvent	A	A	A	A
Ref.	[24]	Ref.	[38]	[38]	[38]	[38]
1	147.4	1	n.d.	190.3	189.9	191.0
2	128.1	2	55.5	139.0	139.0	140.0
3	116.3	3	56.4	141.2	141.4	141.7
4	149.7	4	191.8	185.0	184.4	193.9
5	113.9	5	134.3	134.6	133.3	55.9
6	116.2	6	145.0	144.2	144.0	61.4
1ʹ	28.7	7	49.7	50.5	50.5	48.5
2ʹ	123.5	8	78.0	78.0	78.3	79.3
3ʹ	135.5	9	40.2	40.2	40.5	39.8
4ʹ	38.1	10	21.0	21.1	20.9	21.1
5ʹ	26.8	11	39.3	39.5	39.5	34.4
6ʹ	145.2	12	49.2	49.3	48.9	49.8
7ʹ	134.0	13	83.8	82.8	82.8	83.7
8ʹ	201.8	14	212.2	213.0	212.3	212.2
9ʹ	69.8	15	77.4	77.2	77.4	77.0
10ʹ	122.9	16	118.7	118.6	117.8	118.5
11ʹ	138.6	17	142.3	142.5	143.0	142.7
12ʹ	25.8	18	25.9	25.9	25.6	25.9
13ʹ	15.8	19	27.1	27.1	26.7	27.4
14ʹ	11.8	20	8.7	8.8	8.6	11.0
15ʹ	18.3	21	18.9	18.7	18.6	18.7

**Table 4:** ^13^C chemical shifts of (δ in ppm) longithorones derivatives.

Carbon	23	24	25	26	27	28	29	32	33
Solvent	C	C	C	C	C	A	C	A	A
Ref.	[40]	[40]	[40]	[40]	[40]	[40]	[40]	[41]	[41]
1	27.6	29.1	28.7	29.5	27.8	29.2	29.0	28.2	28.3
2	125.3	122.1	120.9	121.7	125.9	123.9	122.9	122.3	120.5
3	136.3	138.5	139.1	139.6	135.8	136.9	136.0	135.3	137.6
4	40.5	39.0	27.9	39.7	40.1	39.0	42.5	39.5	39.4
5	36.3	24.1	24.1	25.4	37.4	36.8	37.3	23.9	23.9
6	119.7	122.6	123.2	131.5	120.4	121.8	122.0	123.9	123.0
7	134.7	135.2	135.2	131.4	134.4	138.1	137.6	134.9	135.3
8	37.4	39.5	39.7	44.1	37.8	36.1	35.0	39.8	39.6
9	26.6	29.2	30.3	43.5	26.5	26.0	27.4	29.0	28.6
10	126.6	127.4	126.7	126.6	126.6	127.2	126.4	128.2	128.8
11	132.5	130.9	132.1	132.6	132.3	130.9	131.7	133.8	130.5
12	35.6	32.1	32.9	17.7	35.4	31.7	33.1	29.0	36.9
13	15.3	14.6	22.2	15.4	15.1	15.4	15.2	14.9	14.8
14	32.9	15.6	16.0	14.2	37.3	37.4	37.8	16.1	16.2
15	27.6	26.5	27.2	25.9	27.5	26.2	27.1	22.8	22.5
16	149.4	148.3	149.0	151.7	149.5	146.9	148.3	38.0	46.5
17	187.2	187.8	187.2	187.6	186.4	187.4	187.9	71.5	200.7
18	131.5	130.2	132.9	149.3	131.5	129.5	129.8	147.1	136.4
19	148.1	147.8	149.4	129.6	147.8	148.0	148.1	138.4	151.7
20	187.0	188.1	187.4	188.8	187.0	188.1	187.0	196.8	197.1
21	133.6	132.4	133.1	133.1	133.5	132.5	132.5	42.0	42.0
1ʹ	37.8	-	-	-	29.5	28.3	28.7	-	-
2ʹ	30.7	-	-	-	39.2	44.7	39.1	-	-
3ʹ	49.4	-	-	-	52.8	52.5	53.7	-	-
4ʹ	27.6	-	-	-	30.9	30.1	33.1	-	-
5ʹ	38.1	-	-	-	21.1	19.7	22.4	-	-
6ʹ	126.1	-	-	-	130.4	129.2	130.6	-	-
7ʹ	143.8	-	-	-	135.4	135.1	134.6	-	-
8ʹ	34.3	-	-	-	40.5	39.9	39.8	-	-
9ʹ	29.9	-	-	-	32.2	32.1	31.4	-	-
10ʹ	130.4	-	-	-	43.4	43.2	43.3	-	-
11ʹ	132.2	-	-	-	147.3	147.8	147.0	-	-
12ʹ	34.5	-	-	-	111.1	110.9	111.3	-	-
13ʹ	205.5	-	-	-	21.4	206.3	203.8	-	-
14ʹ	30.9	-	-	-	15.4	15.7	14.8	-	-
15ʹ	27.5	-	-	-	204.6	21.4	21.2	-	-
16ʹ	155.7	-	-	-	151.2	151.5	151.8	-	-
17ʹ	200.7	-	-	-	187.0	186.1	187.4	-	-
18ʹ	56.3	-	-	-	151.9	152.2	152.5	-	-
19ʹ	52.9	-	-	-	134.0	133.8	131.8	-	-
20ʹ	201.2	-	-	-	187.1	188.5	187.6	-	-
21ʹ	139.2	-	-	-	130.4	129.9	130.9	-	-
1-OCH_3_		-	-	-	-	-	-	-	-

**Table 5:** ^13^C chemical shifts of (δ in ppm) longithorols and floresolides derivatives.

Carbon	34	35	36	37	38	39	40
Solvent	E	E	F	F	A	A	A
Ref.	[47]	[47]	[48]	[48]	[49]	[49]	[49]
1	67.1	67.2	71.6	81.2	33.9	34.0	30.1
2	129.0	129.6	130.0	127.4	63.7	62.5	137.8
3	135.5	135.1	131.4	133.9	59.1	58.3	136.1
4	39.9	40.4	38.5	38.5	33.4	32.6	31.4
5	37.4	38.4	23.9	23.8	24.0	23.2	25.0
6	119.2	119.6	120.8	121.0	123.4	126.7	124.6
7	137.6	137.2	134.1	134.2	137.4	141.2	135.8
8	36.7	37.3	38.9	38.8	39.3	35.4	37.4
9	24.6	24.7	25.4	26.0	29.6	25.0	29.3
10	126.6	126.5	125.7	125.8	43.2	44.4	41.5
11	131.5	131.4	133.1	133.0	149.3	143.9	149.2
12	36.8	36.8	33.2	32.9	109.4	111.8	108.9
13	16.3	16.2	15.3	15.1	21.9	22.5	22.4
14	39.1	37.8	16.4	16.1	14.9	58.3	13.6
15	27.5	27.5	27.4	27.3	169.1	167.8	166.6
16	133.7	133.7	125.7	126.3	137.3	128.8	135.7
17	148.0	148.0	147.4	147.1	142.7	142.9	144.2
18	120.9	120.8	112.4	114.3	128.9	133.7	132.7
19	132.8	132.7	127.5	124.8	114.0	112.2	112.6
20	143.6	143.6	147.4	147.4	153.0	150.1	152.6
21	126.3	126.2	117.1	117.2	114.2	109.5	112.1
1ʹ	31.6	32.6	-	-	-	-	-
2ʹ	44.4	39.4	-	-	-	-	-
3ʹ	53.1	52.9	-	-	-	-	-
4ʹ	25.8	30.6	-	-	-	-	-
5ʹ	19.9	20.9	-	-	-	-	-
6ʹ	126.0	126.4	-	-	-	-	-
7ʹ	133.9	133.9	-	-	-	-	-
8ʹ	40.7	40.6	-	-	-	-	-
9ʹ	31.1	31.3	-	-	-	-	-
10ʹ	45.6	45.8	-	-	-	-	-
11ʹ	149.5	149.3	-	-	-	-	-
12ʹ	109.8	110.0	-	-	-	-	-
13ʹ	208.7	205.9	-	-	-	-	-
14ʹ	15.4	15.2	-	-	-	-	-
15ʹ	22.4	22.5	-	-	-	-	-
16ʹ	138.4	138.1	-	-	-	-	-
17ʹ	143.0	142.8	-	-	-	-	-
18ʹ	136.6	136.1	-	-	-	-	-
19ʹ	120.8	120.8	-	-	-	-	-
20ʹ	148.7	148.9	-	-	-	-	-
21ʹ	119.4	119.4	-	-	-	-	-
1-OCH_3_	-	-	-	55.4	-	-	-

**Table 6:** ^13^C chemical shifts (δ in ppm) of scabellone derivatives.

Carbon	41	Carbon	42	Carbon	43	44
Solvent	A	Solvent	A	Solvent	A	A
Ref.	[17]	Ref.	[17]	Ref.	[17]	[17]
1	186.4	1	182.6	1	185.4	184.9
2	107.6	2	107.3	2	107.1	107.2
3	158.3	3	157.8	3	158.3’	158.0
4	181.5	4	178.7	4	179.0	178.7
5	145.7	4a	130.8	4a	131.4	131.1
6	138.4	5	67.6	5	67.7	67.7
7	109.1	6a	151.4	6a	151.6	151.3
8	145.7	7	98.4	7	101.2	101.0
9	101.4	8	150.0	8	153.0	152.5
10	149.4	9	139.2	8a	138.3	137.9
10a	136.2	10	126.8	10	77.8	77.4
12	77.5	10a	111.0	11	126.6	127.4
13	131.3	10b	137.6	12	123.8	123.6
14	120.1	1ʹ	116.9	12a	120.2	120.5
14a	120.4	2ʹ	144.3	12b	107.6	107.1
15	25.4	3ʹ	39.7	12c	133.9	133.6
1ʹ	26.6	4ʹ	26.3	13	24.5	25.9
2ʹ	118.0	5ʹ	123.6	1ʹ	117.0	117.0
3ʹ	138.1	6ʹ	131.7	2ʹ	144.3	144.0
4ʹ	39.2	7ʹ	17.6	3ʹ	39.7	39.4
5ʹ	26.3	8ʹ	25.6	4ʹ	26.2	26.1
6ʹ	124.1	9ʹ	17.2	5ʹ	123.6	123.7
7ʹ	131.2	1ʹʹ	26.5	6ʹ	131.7	131.5
8ʹ	17.3	2ʹʹ	124.2	7ʹ	17.7	17.6
9ʹ	25.4	3ʹʹ	137.1	8ʹ	25.6	25.8
10ʹ	15.6	4ʹʹ	39.8	9ʹ	17.2	17.2
1ʹʹ	39.6	5ʹʹ	26.2	1ʹʹ	41.1	38.5
2ʹʹ	22.2	6ʹʹ	123.8	2ʹʹ	23.2	22.4
3ʹʹ	124.0	7ʹʹ	131.6	3ʹʹ	124.2	124.3
4ʹʹ	131.5	8ʹʹ	17.5	4ʹʹ	131.7	131.5
5ʹʹ	17.3	9ʹʹ	25.5	5ʹʹ	17.7	17.6
6ʹʹ	25.4	10ʹʹ	16.5	6ʹʹ	25.7	25.8
3-OCH_3_	56.2	3-OCH_3_	56.1	3-OCH_3_	56.2	56.5
10-OCH_3_	56.2	8-OCH_3_	56.1	8-OCH_3_	56.2	56.5

The presence in the ^13^C-NMR data of 4 olefinic resonances for two trisubstituted double bonds clearly suggests a linear diprenyl chain in **2**, **3**, **6**, **7**, **10**, and **17**. The configurations at C-2ʹ and C-3ʹ for the linear diprenyl chain (as in **2**, **3**, **4**, **6**, **7**, **10**, **11**, and **17**) can be established on the bases of the relatively low-field and high-field signals for the C-10ʹ of the (*Z*) compound and the sterically more congested (*E*) compound, respectively [15,18]. Thus, this δ value allows for the determination of whether the diprenylated substituent is a neryl or geranyl group.

**Table 7:** ^13^C chemical shifts (δ in ppm) of conicaquinones, aplidinones, thiaplidiaquinones, and conithiaquinones.

Carbon	45	46	Carbon	47	48	49	Carbon	50	51	Carbon	52	53
Solvent	A	A	Solvent	A	A	D	Solvent	A	A	Solvent	D	D
Ref.	[53]	[53]	Ref.	[54]	[54]	[54]	Ref.	[55]	[55]	Ref.	[58]	[58]
1	25.3	25.7	2	49.0	48.6	49.0	1	111.4	113.8	2	48.3	48.2
2	135.6	133.3	3	40.5	40.1	41.0	2	149.7	148.9	3	39.4	39.3
3	122.9	122.2	4a	144.3	149.5	151.0	3	120.0	133.4	4a	146.1	146.4
4	28.7	29.3	5	179.3	173.1	174.5	4	132.3	118.4	5	181.5	181.5
5	34.8	36.5	6	126.7	108.9	110.0	4a	145.9	148.1	5a	148.0	150.0
6	147.3	151.5	7	157.3	147.8	149.0	6	67.2	67.4	6	37.5	37.5
7	135.5	133.2	8	174.2	175.4	176.6	6a	138.6	137.4	6a	47.5	48.1
8	125.8	128.2	8a	109.2	109.2	109.0	7	175.1	175.2	7	21.1	21.7
9	130.6	127.1	1ʹ	22.2	23.4	24.0	7a	110.1	109.9	8	33.1	33.7
10	174.9	178.4	2ʹ	119.2	119.5	124.8	9	48.6	48.6	9	82.7	82.4
11	146.7	113.4	3ʹ	137.6	138.4	137.0	10	39.9	39.8	9a	51.8	52.1
12	113.4	146.7	4ʹ	39.7	39.5	40.5	11a	144.0	144.3	10	65.1	74.4
13	178.9	174.8	5ʹ	26.6	26.3	27.8	12	179.1	179.2	10a	147.5	149.7
14	129.3	132.6	6ʹ	124.1	123.8	125.0	12a	128.0	128.0	11	180.5	179.7
15	126.0	126.5	7ʹ	131.5	131.8	133.0	12b	116.6	114.4	11a	110.0	110.3
16	17.5	17.6	8ʹ	25.6	25.7	26.1	1ʹ	28.1	29.1	12-CH_3_	21.2	21.6
17	39.8	48.1	9ʹ	16.1	16.2	16.5	2ʹ	121.3	120.5	13-OCH_3_	48.5	48.5
18	48.2	39.8	10ʹ	17.7	17.7	17.8	3ʹ	136.8	138.7	16-OCH_3_	-	58.0
-	-	-	1ʹʹ	-	-	41.5	4ʹ	39.7	39.8	14-CH_3_	18.6	18.4
-	-	-	2ʹʹ	-	-	51.5	5ʹ	26.6	26.6	15-CH_3_	25.4	25.7
-	-	-	OCH_3_	62.3	-	-	6ʹ	124.1	123.9	-	-	-
-	-	-	-	-	-	-	7ʹ	131.4	131.8	-	-	-
-	-	-	-	-	-	-	8ʹ	25.7	25.7	-	-	-
-	-	-	-	-	-	-	9ʹ	16.1	16.2	-	-	-
-	-	-	-	-	-	-	10ʹ	16.1	17.7	-	-	-
-	-	-	-	-	-	-	1ʹʹ	116.8	116.8	-	-	-
-	-	-	-	-	-	-	2ʹʹ	145.3	145.0	-	-	-
-	-	-	-	-	-	-	3ʹʹ	39.7	39.7	-	-	-
-	-	-	-	-	-	-	4ʹʹ	26.2	26.2	-	-	-
-	-	-	-	-	-	-	5ʹʹ	123.5	123.5	-	-	-
-	-	-	-	-	-	-	6ʹʹ	131.7	131.8	-	-	-
-	-	-	-	-	-	-	7ʹʹ	25.6	25.6	-	-	-
-	-	-	-	-	-	-	8ʹʹ	17.3	17.4	-	-	-
-	-	-	-	-	-	-	9ʹʹ	17.5	17.7	-	-	-

The lack of ^13^C-NMR resonance for the group of unsaturated carbon atoms, which were substituted by a δ-deshielded value (δ_C_ 70.9), as in **4** [15], suggest the presence of a hydroxyl group. This finding was also observed for compounds **11** and **12**.

Conidinone (**8**), which has a cyclic diprenyl substituent, presented a 1H-NMR spectrum with signals at δ_H_ 6.71 (1H, d, *J* = 10.3 Hz), δ_H_ 6.87 (1H, d, *J* = 10.3 Hz), δ_H_ 2.88 (1H, d, *J* = 16.7 Hz), and δ_H_ 2.81 (1H, d, *J* = 16.7 Hz), defining the presence of a 2,2-disubstituted cyclohexenedione ring in the molecule. The ^13^C-NMR of **8** includes two α,β-unsaturated ketone carbonyl signals, as well as a trisubstituted and a disubstituted double bond [21].

Compound **15** and **16** presented a substituted hydroquinone nucleus, together with a 3,4-disubstituted-1-methylcyclohexene ring, and 1-hydroxyisopropyl unit, which was attached to C-6ʹ of the cyclohexene ring upon observation of the ROESY spectrum and NOE experiment, respectively. The difference between **15** and **16** was the presence of a methoxy group at **16** [31,35].

Compound **17** produced ^1^H-NMR spectrum resonances attributable to two aromatic protons at δ_H_ 6.75 (1H, d, *J* = 2.1 Hz, H-5) and δ_H_ 6.54 (1H, d, *J* = 2.1 Hz, H-3), two olefinic protons at δ_H_ 5.29 (1H, t, *J* = 6.6 Hz, H-2′) and δ_H_ 5.09 (1H, t, *J* = 6.6 Hz, H-6′), three moderately deshielded methylene signals (δ_H_ 3.27, 2.08, and 2.00), one methoxyl signal at δ_H_ 3.83, and three allylic methyl singlets at δ_H_ 1.69, δ_H_ 1.65, and δ_H_ 1.58. Direct comparison of the ^1^H and ^13^C chemical shifts of the aromatic ring signals of **17** with a synthetic derivative of **17** (compound which had a hydroxyl group at C-4 instead of the sulfate) allow Chan and co-worker to identified an upfield shift of C-4 and downfield shifts of C-3 and C-5 in the ^13^C-NMR spectrum and downfield shifts of H-3 and H-5 in the ^1^H-NMR spectrum which were consistent with the placement of the sulfate group at the C-4 [17].

The ^13^C-NMR data for Rossinone A (**18**), Table 3, allowed for the identifications of a triprenylated (farnesyl) hydroquinone-bearing substitution in the terminal prenyl unit. A α-hydroxy ketone group (δ_C_ 201.8) and a carbinol resonance at δ_C_ 69.8 in the side chain of **18** were established [23].

The Rossinone family, mainly compounds **19**–**22**, have a cyclic triprenylated group. When comparing **19** and **20**, the principal differences were in the chemical shift of the AB quartet due to the quinone protons resonating at higher fields (H-2: δ_H_ 3.75 (d, *J* = 3.5 Hz) in **19** and δ_H_ 6.79 (d, *J* = 10.5 Hz) in **20**; H-3: δ_H_ 3.80 (d, *J* = 3.5 Hz) in **19** and δ_H_ 6.90 (d, *J* = 10.5 Hz) in **20**). Additionally, the ^13^C-NMR spectrum of **19** contained signals at δ_C_ 55.5 (C-2) and δ_C_ 56.4 (C-3) in the place of the quinone resonances of **20** observed at δ_C_ 139.0 (C-2) and δ_C_ 141.2 (C-3) [38].

The compound 3-*Epi*-rossinone B (**21**) was an isomer of rossinone B (**20**). The analysis of the ^1^H-NMR revealed significant differences in the proton values of both H-7 and H-11 (H-7: δ_H_ 2.06 in **20** and δ_H_ 2.48 in **21**; H-11: δ_H_ 2.67 in **20** compared to δ_H_ 2.10 in **21**) that could be explained by the diverse steric influence of the hydroxyl group in these two compounds; the structure was confirmed by NOE experiments [38].

For 5,6-Epoxy-rossinone B (**22**), revealed in ^1^H-NMR spectrum the lack of the double bond in ring B when compared with **20**, which is substituted by an epoxide ring in **22**. The H-6 was attributed to a singlet at δ_H_ 3.80. In the ^13^C-NMR spectrum the values at δ_C_ 61.4 and δ_C_ 55.7, were assigned to C-6 and C-5, respectively. Additionally, due to the absence of the conjugated double bond, the C-4 quinone carbonyl in **22** was shifted down-field (δ_C_ 193.9) with respect to **20** (δ_C_ 185.0) [38].

The complex and unprecedented structure of longithorone A (**23**) was determined by crystal X-ray diffraction, while the enantioselective biomimetic synthesis of longithorone A was accomplished by Layton and coworkers [59]. The ^1^H and ^13^C-NMR of (**23**) (Table 4) revealed that **23** contained eight double bonds and five carbonyl groups, including one aldehyde (δ_H_ 9.6 and δ_C_ 206.6), in accord with the presence of seven carbocycles to satisfy the unsaturation number revealed by the mass spectral data (C_42_H_46_O_5_) [42].

Longithorones B (**24**) and C (**25**) are optically active isomers with the double bond configurations 2*E* and 2*Z* in **24** and **25**, respectively. The singlets at δ_H_ 6.23 (H-18) and δ_H_ 6.45 (H-21) for **24** and δ_H_ 6.38 (H-18 and H-21) for **25** are representative of *para*-disubstituted benzoquinone core. The synthesis of Longithorone B was accomplished by Kato *et al.* [60].

The structures of longithorones E (**27**) and F (**28**) are very similar. However, the NOESY experiment for Longithorone F revealed that H-13 is spatially close to H-6, H-18, H-1α, and H-4a. On the other hand, the NOE experiment of **27** showed interactions between H-13 with H-6 and H-1β, but not with H-18. Thus, the dimeric longithorones E (**27**) and F (**28**) are atropoisomers with respect to the *para*-disubstituted benzoquinone ring [40]. In turn, longithorone F (**28**) differs from longithorone G (**29**) in its C-2ʹ, C-3ʹ, and C-10ʹ stereochemistry. The NOESY data for **29** revealed correlations between the aldehyde hydrogen H-13 with H-5 and H-2ʹ suggesting that these protons were all on the same face of the molecule. These correlations were not, however, observed in the NOE experiment for **28**.

The farnesyl chains in longithorone J (**32**) and K (**33**) were characterized by three shielded olefinic methyl resonances, two of these (δ_C_ 14.9 and δ_C_ 16.9) assigned *E*-geometry configuration and the other (δ_H_ 22.8) was assigned a *Z*-geometry. The ^1^H and ^13^C-NMR spectra of longithorone J include an oxymethine proton at δ_H_ 4.84 (δ_C_ 71.5) in contrast to the presence of two ketone resonances at δ_C_ 200.7 and δ_C_ 196.8 in longithorone K (**33**). The absolute stereochemistry of longithorone J (**32**) has been determined by the advanced Mosher method, while the absolute stereochemistry for longithorone K (**33**) was suggested by comparison with **32** and based on biosynthesis [43].

A comparison of the ^1^H and ^13^C-NMR data for longithorols A–D (**34**–**37**) (Table 5) with previous longithorones reveals that, in longithorols, signals for two substituted 1,4-hydroquinones (C-16 to C-21 and C-16ʹ to C-21ʹ) are present instead of signals for two substituted 1,4-quinones. In addition, an acetoxymethine group can be recognized by the signals at δ_H_ 6.61 (d, *J* = 10.0 Hz) and δ_C_ 67.1 (for example, in **34**), which replace the methylene group corresponding to C-1 in longithorones [47]. The absolute stereochemistry of longithorol C (**36**) has been determined by the advanced Mosher method [48].

Floresolides skeleton can be recognized by the presence of an endocyclic ε-lactone with an α,β-epoxy-group (δ_C_ 63.7 and δ_C_ 59.1) in **38** and (δ_C_ 62.5 and δ_C_ 58.3) in **39**, and a double bond at the C-2 and C-3 position (δ_C_ 137.8 and δ_C_ 136.1) in **40** (Table 5). The terminal methylene group at the C-11 and C-12 positions is also typical in this structural family. The Floresolide **39** has a primary alcohol and a fully substituted benzene ring including two bromine atoms in its structure. Compound **40** has absolute stereostructure, confirmed by crystal-X-ray diffraction [49].

Scabellones are conspicuous for the rare tricycle benzo[*c*] chromene-7,10 dione core in their structure. The signals at δ_C_ 182.6 (C-1) and δ_C_ 178.7 (C-4) (Table 6) define the presence of the quinonoid ring system, while the HMBC correlations from the aromatic proton resonance at δ_H_ 6.40 (H-7) to the ^13^C resonances at δ_C_ 150.0 (C-8), δ_C_ 139.2 (C-9), δ_C_ 151.4 (C-6a) and δ_C_ 111.0 (C-10a), together with the methoxyl group at δ_H_ 3.89 (C-8) and the signal at δ_C_ 126.8 (C-10), define the tetrasubstituted phenol in Scabellone B (**42**), the active representative compound of this family [17].

The structures of conicaquinones (**45**–**46**), aplidinones (**47**–**49**), thiaplidiaquinones (**50**–**51**) and conithiaquinones (**52**–**53**) have the presence of an unusual 1,1-dioxo-1,4-thiazine ring characterized by N-H (δ_H_ 6.78), two methylene protons at δ_H_ 4.10 (δ_C_ 39.8) and δ_H_ 3.36 (δ_C_ 48.2) in common, as exemplified in Conicaquinone A (**45**) (Table 7) [53]. In turn, the aplidinones contain a geranyl chain that is attached to the system -NHCH_2_CH_2_SO_2_^−^ in addition to the quinone ring. Finally, the thiaplidiaquinones and conithiquinones contain a tetracyclic skeleton. Thiaplidiaquinones have the same tricyclic 6H-benzo[*c*] chromene-7,10-dione described for Scabellone B (**42**) in their structure [17]. On the other hand, the signals related to C-6 to C-10 in ^13^C-NMR spectra, in addition to the signal of the methoxyl group (C-13), the methyl protons C-12, C-14 and C-15 and the signal of hydroxy group positioned at C-10 in the ^1^H-NMR spectra, establish the presence of C and D ring fused at 1,4-benzoquinone/1,1-dioxo-1,4-thiazide bicyclic system (A and B rings) of conithiaquinone skeleton [58].

## 2. Conclusions

The studies presented in this review reveal the importance of the prenylated quinone and hydroquinone metabolites, which have important biological activities and occur with frequency in ascidians of the genus *Aplidium*. Those non-nitrogenous metabolites, mainly prenyl quinones or hydroquinones, which can be either linear or cyclic compounds, such as rossinones, longithorones, longithorols, floresolides, scabellones, conicaquinols, aplidinones, thiaplidiaquinones, and conithiaquinones, are examples of meroterpenes. The evaluated compounds mainly presented cytotoxic, anti-inflammatory, and antimicrobial activities. Furthermore, ascidians (tunicates) are a promising source of new bioactive compounds from marine environments [61,62,63]. The *Aplidium* genus is able to produce meroterpenes with a large range of structural variety, of which the longithorone series is notable for containing the most complex structures with a metacyclophane and paracyclophane scaffolds. The complex and elaborate structure of some of these meroterpenoids has led several research groups to engage in performing the total synthesis of specific compounds for their structural confirmation and to clarify biosynthetic pathways. The ^13^C-NMR data of a given compound are an important tool for natural products research because it is sometimes possible to propose the structure of a novel natural compound by performing comparisons with data for known compounds. For example, the structure of rossinones, were deduced by comparing 2D NMR spectrum with those from literature data reported for rossinone B. Therefore, considering the enormous diversity in their chemical structures and the biological potential of the prenyl quinones and hydroquinones found in *Aplidium*, it is important to conduct further studies of this genus using a multidisciplinary approach.
